# Which quantitative perfusion estimation model is better at diagnosing myocardial ischaemia? A CE-MARC sub-study

**DOI:** 10.1186/1532-429X-16-S1-P209

**Published:** 2014-01-16

**Authors:** John D Biglands, Derek R Magee, Steven Sourbron, Sven Plein, John P Greenwood, Aleksandra Radjenovic

**Affiliations:** 1 Division of Medical Physics, Leeds Teaching Hospitals, Leeds, UK; 2 School of Computing, University of Leeds, Leeds, UK; 3 MCRC & LIGHT, University of Leeds, Leeds, UK; 4 Institute of Cardiovascular & Medical Sciences, BHF Glasgow Cardiovascular Research Centre, University of Glasgow, Glasgow, UK

## Background

There are multiple methods for quantifying myocardial blood flow from dynamic contrast enhanced MRI (DCE-MRI) cardiac perfusion data sets. Currently there is no documented evidence to suggest the superiority of any of these models for diagnosing myocardial ischaemia. The aim of this study was to compare the diagnostic performance of four such methods.

## Methods

This was a retrospective sub-study using data from the CE-MARC trial (Greenwood et al., Lancet, 2012). A 50 patient sample of patients were selected such that the distribution of risk factors and disease status within the sample was representative of the full CE-MARC cohort. Quantitative myocardial blood flow (MBF) estimates were obtained from the MRI data using four previously proposed models, commonly used in the quantitative cardiac perfusion literature. These models were: Fermi-constrained deconvolution, model independent deconvolution, the uptake model and the one compartment model. Myocardial Perfusion Reserve (MPR) ratios were calculated from the ratio of stress to rest MBF estimates. The presence of myocardial ischaemia was assessed using the consensus diagnosis of invasive, quantitative X-ray angiography and myocardial Single Photon Computed Tomography (SPECT) imaging. This provided a unique gold-standard combining independent anatomical and functional diagnostic measures. Receiver Operator Characteristic (ROC) curves were generated for each perfusion model using 1) the MPR, and 2) the stress MBF as the diagnostic measure. A DeLong, DeLong, Clarke-Pearson comparison was used to test for statistically significant differences in the Area Under the Curve (AUC) values of the four models.

## Results

**Figure 1:**
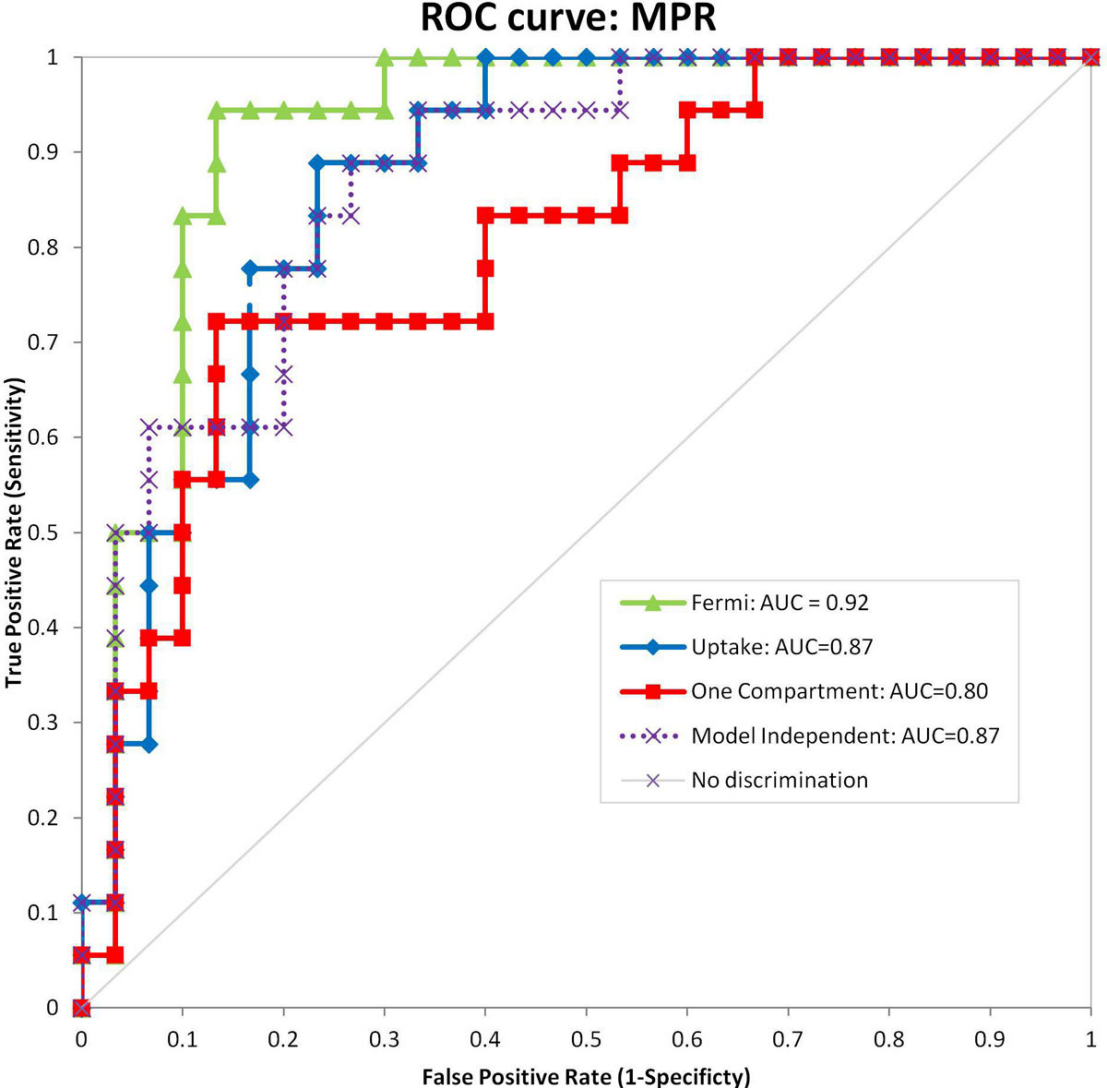
**ROC curves showing diagnostic performance of the four models using MPR as the diagnostic measure, with AUC scores shown in the legend**.

**Figure 2:**
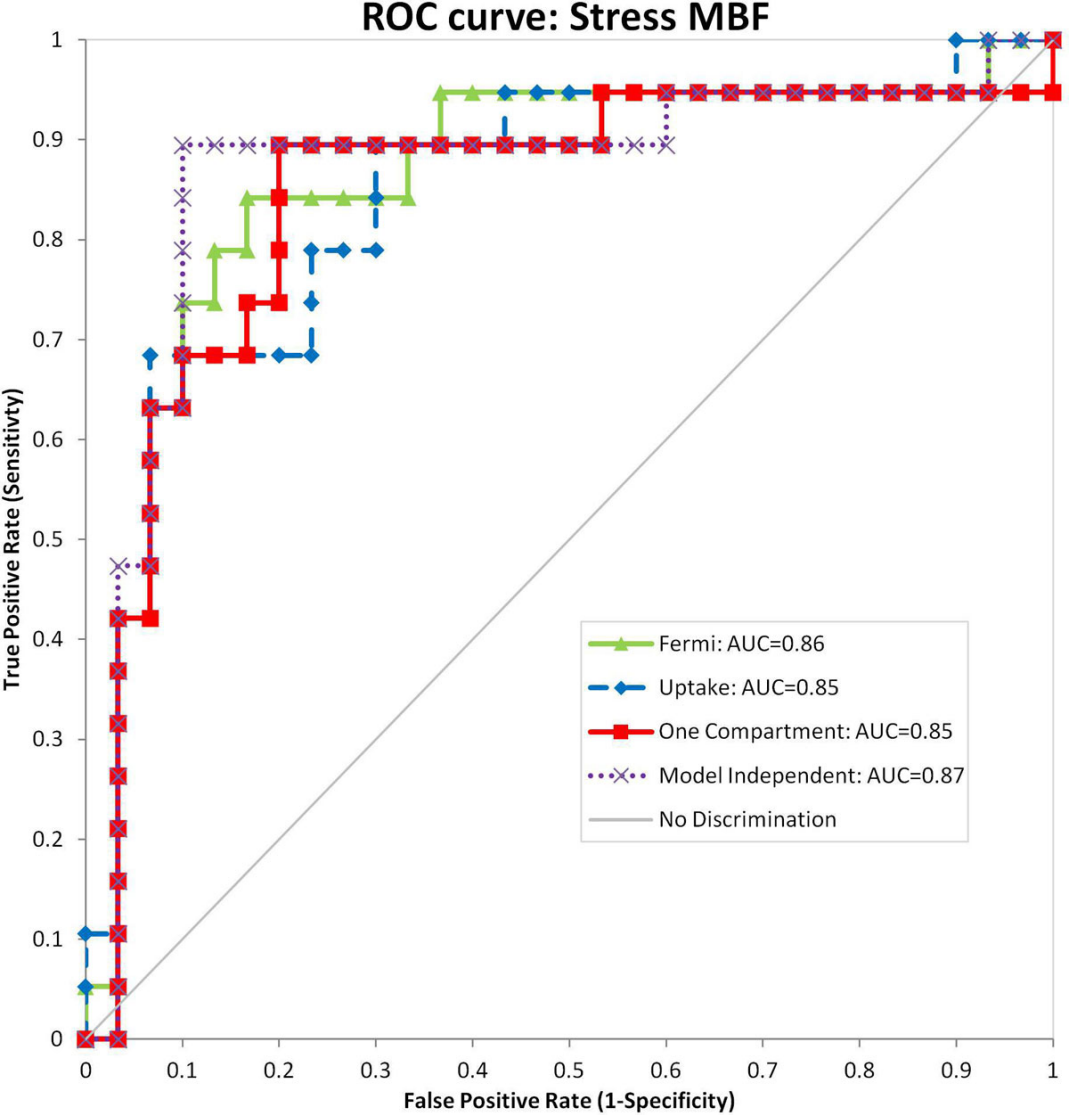
**ROC curves showing diagnostic performance of the four models using stress MBF as the diagnostic measure, with AUC scores shown in the legend**.

The MBF estimates between the models were well correlated with all inter-model comparisons achieving a Pearsons r-value > 0.88. There were almost no significant differences between the diagnostic performances of the four models using either MPR (Figure 1) or stress MBF (Figure 2) as the diagnostic measure. The single exception was the one compartment model based MPR values, which significantly underperformed compared to Fermi when evaluated in terms of MPR (p = 0.02). However, there was no significant difference between the one compartment model and model independent or uptake models. Furthermore no differences were observed with the one compartment model when stress MBF was used as the diagnostic measure.

## Conclusions

There is no evidence to show that any of the models are superior in the diagnosis of myocardial ischaemia. However, the one compartment model should be avoided when using MPR as the diagnostic measure.

## Funding

Theis work was funded by an NIHR doctoral training fellowship.

